# Key Role of Mfd in the Development of Fluoroquinolone Resistance in *Campylobacter jejuni*

**DOI:** 10.1371/journal.ppat.1000083

**Published:** 2008-06-06

**Authors:** Jing Han, Orhan Sahin, Yi-Wen Barton, Qijing Zhang

**Affiliations:** Department of Veterinary Microbiology and Preventive Medicine, College of Veterinary Medicine, Iowa State University, Ames, Iowa, United States of America; Massachusetts General Hospital, United States of America

## Abstract

*Campylobacter jejuni* is a major food-borne pathogen and a common causative agent of human enterocolitis. Fluoroquinolones are a key class of antibiotics prescribed for clinical treatment of enteric infections including campylobacteriosis, but fluoroquinolone-resistant *Campylobacter* readily emerges under the antibiotic selection pressure. To understand the mechanisms involved in the development of fluoroquinolone-resistant C*ampylobacter*, we compared the gene expression profiles of *C. jejuni* in the presence and absence of ciprofloxacin using DNA microarray. Our analysis revealed that multiple genes showed significant changes in expression in the presence of a suprainhibitory concentration of ciprofloxacin. Most importantly, ciprofloxacin induced the expression of *mfd*, which encodes a transcription-repair coupling factor involved in strand-specific DNA repair. Mutation of the *mfd* gene resulted in an approximately 100-fold reduction in the rate of spontaneous mutation to ciprofloxacin resistance, while overexpression of *mfd* elevated the mutation frequency. In addition, loss of *mfd* in *C. jejuni* significantly reduced the development of fluoroquinolone-resistant *Campylobacter* in culture media or chickens treated with fluoroquinolones. These findings indicate that Mfd is important for the development of fluoroquinolone resistance in *Campylobacter*, reveal a previously unrecognized function of Mfd in promoting mutation frequencies, and identify a potential molecular target for reducing the emergence of fluoroquinolone-resistant *Campylobacter*.

## Author Summary

As a food-borne bacterial pathogen, *Campylobacter jejuni* is a common causative agent of gastrointestinal illnesses in humans. Development of antibiotic resistance in *Campylobacter*, especially to fluoroquinolone (a broad-spectrum antimicrobial), compromises clinical treatments and presents a major public health threat. It is not well understood why *Campylobacter* is highly adaptable to fluoroquinolone treatment or how it acquires mutations associated with fluoroquinolone resistance. Understanding the molecular mechanisms involved in the resistance development will help us to reduce the emergence of fluoroquinolone-resistant *Campylobacter*. Using DNA microarray and other molecular methods, as well as animal studies, we uncovered the key role of Mfd in promoting spontaneous mutations and development of fluoroquinolone resistance in *Campylobacter*. Mfd is a transcription-repair coupling factor involved in DNA repair and was not previously known for its role in promoting mutations conferring antibiotic resistance. Our findings not only reveal a novel function of Mfd, but also provide a potential molecular target for reducing the emergence of fluoroquinolone-resistant *Campylobacter*.

## Introduction

*Campylobacter jejuni*, a Gram-negative microaerobic bacterium, is one of the most prevalent bacterial foodborne pathogens in humans, causing more than 2 million cases of diarrhea each year in the U.S. alone [1],[2],[3]. As an enteric pathogen, this organism causes watery diarrhea and/or hemorrhagic colitis. *Campylobacter* infection is also the most common antecedent to Guillain-Barre syndrome, an acute flaccid paralysis that may lead to respiratory muscle compromise and death [4],[5]. In developed countries, person-to-person transmission of *Campylobacter* is rare, and the main source of human *Campylobacter* infections is via food, water, or milk contaminated by *Campylobacter* [6].

Fluoroquinolone (FQ) antimicrobials are often prescribed for clinical treatment of diarrhea caused by enteric bacterial pathogens including *Campylobacter* [7],[8]. However, *Campylobacter* is increasingly resistant to FQ antimicrobials, which has become a major concern for public health [9],[10],[11]. FQ-resistant (FQ^R^) *Campylobacter* developed in food producing animals can be transmitted to humans via the food chain. Poultry are considered the major reservoir for *C. jejuni* and a significant source for FQ^R^ *Campylobacter* infections in humans, because the majority of domestically acquired cases of human campylobacteriosis result from consumption of undercooked chicken or food contaminated by raw chicken [2],[12],[13]. Although FQ antimicrobials have been banned since 2005 in poultry production in the U.S., FQ^R^ *Campylobacter* continue to persist on poultry farms [14],[15],[16].

The main targets of FQs in bacteria are DNA gyrases and/or topoisomerase IV [17],[18]. In *Campylobacter*, the resistance to FQ antimicrobials is mediated by point mutation in the quinolone resistance-determining region (QRDR) of *gyrA* in conjunction with the function of the multidrug efflux pump CmeABC [10],[19],[20],[21]. Acquisition of high-level FQ resistance in *Campylobacter* does not require stepwise accumulation of point mutations in *gyrA*. Instead, a single point mutation in *gyrA* can lead to clinically relevant levels of resistance to FQ antimicrobials [19],[20],[22]. Specific mutations at positions Thr-86, Asp-90 and Ala-70 in GyrA have been linked to FQ resistance in *C. jejuni* [10],[19]. When enumerated by ciprofloxacin (CIPRO)-containing plates, spontaneous FQ^R^ *Campylobacter* mutants occur at a frequency as high as 10^−6^ [23], suggesting that *C. jejuni* possess a high mutation rate to FQ resistance. CmeABC, an energy-dependent efflux system, contributes significantly to the intrinsic and acquired resistance to FQs in *C. jejuni* by reducing the accumulation of the antibiotics within *Campylobacter* cells [19],[20],[24],[25]. The expression level of *cmeABC* also influences the frequencies of emergence of spontaneous FQ^R^ mutants [23].

One unique feature of FQ resistance development in *Campylobacter* is the rapid emergence of FQ^R^ mutants from a FQ-susceptible population when treated with FQ antimicrobials. This has been observed in *Campylobacter-*infected animals or patients treated with FQs [19],[26],[27],[28],[29],[30]. In chickens infected with FQ-susceptible *Campylobacter*, treatment with enrofloxacin resulted in the emergence of FQ^R^ *Campylobacter* mutants that were detected in feces within 24–48 hours after the initiation of treatment, and the FQ^R^ population continued to expand during the treatment and eventually occupied the intestinal tract at a density as high as 10^7^ CFU/g feces [19],[29],[30]. As shown in a comparison study, the same FQ treatment did not result in the development and enrichment of FQ^R^ *E. coli* in chickens [29], suggesting that *C. jejuni* has a unique ability to adapt to FQ treatment. This high frequency of emergence of FQ^R^ *Campylobacter* mutants in response to the selection pressure may have directly contributed to the global prevalence of FQ^R^ *Campylobacter*. For example, multiple studies have shown the temporal link between the approval of FQ antimicrobials for use in animal production and the rapid increase of FQ^R^ *Campylobacter* isolates from both animals and humans [9],[31],[32],[33],[34],[35],[36],[37],[38],[39]. In some regions of the world, the vast majority of *Campylobacter* isolates have become resistant to FQ antimicrobials [22],[40],[41].

The rapidness and magnitude of FQ resistance development in *Campylobacter* in response to FQ treatment suggest that both selective enrichment of pre-existing spontaneous mutants and adaptive gene expression may contribute to the emergence of FQ^R^ *Campylobacter,* but how *Campylobacter* responds to FQ treatment is unknown. Within bacterial cells, FQ antimicrobials form a stable complex with gyrases and DNA, which generates double-stranded breaks in DNA and leads to bacterial death [18]. In other bacteria, antibiotic treatments (including FQs) induce the SOS response, which upregulates multiple genes involved in DNA repair, recombination, and mutation as well as other functions [42],[43],[44],[45]. The SOS response is controlled by LexA, a transcriptional repressor. DNA damage triggers LexA autocleavage, which derepresses the SOS genes controlled by LexA. Once activated, SOS response promotes the development of drug resistance, horizontal transfer of genetic materials, and production of virulence factors [45],[46],[47]. Unlike many other bacterial organisms, *epsilonproteobacteria* including *Campylobacter* and *Helicobacter* don't have a LexA ortholog [46] and also lack many genes involved in DNA repair, recombination, and mutagenesis, such as the *mutHL* genes (methyl-directed mismatch repair), the *umuCD* genes (UV-induced mutagenesis), and SOS-controlled error-prone DNA polymerases [48],[49],[50]. These observations suggest that *Campylobacter* may not have the typical SOS response system. In light of this possibility, it is intriguing to determine how *Campylobacter* copes with FQ treatment and what facilitates the emergence of FQ^R^ mutants in *Campylobacter*.

In this study, we examined the gene expression profiles of *C. jejuni* NCTC 11168 in response to treatment with CIPRO. Consistent with the prediction, a typical SOS response was not observed in *Campylobacter* treated with CIPRO. However, 45 genes showed ≥1.5-fold (p<0.05) changes in expression when *Campylobacter* was exposed to a suprainhibitory dose of CIPRO for 30 min. One of the up-regulated genes was *mfd* (mutation frequency decline), which encodes a transcription-repair coupling factor involved in DNA repair. The *mfd* gene in *E. coli* was originally linked to the phenotype of mutation frequency decline [51],[52]. Subsequently it was found that Mfd functions as a transcription-repair coupling factor and promotes strand-specific DNA repair [53],[54]. DNA lesions stall RNA polymerase during transcription. Mfd displaces the stalled RNA polymerase from the DNA lesions in an ATP-dependent manner, recruits the UvrABC excinuclease complex, and enhances the repair of the DNA lesions on the transcribed strand [54],[55]. Thus, Mfd couples transcription with DNA repair and contributes to mutation frequency decline. Recently it was reported that depending on the nature of DNA damage and the availability of NTPs, Mfd can also promote the forward translocation of arrested RNA polymerase in the absence of repair, leading to transcriptional bypass of non-repaired lesions [55]. In contrast to its previously known function in the decline of mutation frequency in other bacterial organisms [51],[52], Mfd in *Campylobacter* was found to promote the emergence of spontaneous FQ^R^ mutants and the development of FQ^R^ mutants under FQ treatments in this study. These findings define a novel function of Mfd and significantly improve our understanding of the molecular mechanisms underlying the development of FQ^R^ *Campylobacter*.

## Results

### Transcriptional analysis of *C. jejuni* response to FQ treatment

To understand the adaptive response of *Campylobacter* to FQ treatment, DNA microarray was used to analyze the transcriptional changes in *C. jejuni* NCTC 11168 after exposure to CIPRO. When the *Campylobacter* cells were treated with a subinhibitory concentration (0.06 µg/ml; 0.5× the MIC) of CIPRO for 1.5 hours, no genes showed ≥1.5-fold changes in expression, suggesting that the transcriptional response to the low dose of CIPRO was very limited. When the *Campylobacter* cells were treated with a suprainhibitory concentration (1.25 µg/ml; 10× the MIC) of CIPRO for 30 min, 45 genes showed ≥1.5-fold (*p*<0.05) changes in expression (Table 1), among which 13 were up-regulated and 32 were down-regulated. The up-regulated genes are involved in cell membrane biosynthesis, cellular processes, and transcription-coupled DNA repair or have unknown functions, while the majority of the down-regulated genes are involved in energy metabolisms (Table 1). Consistent with the lack of LexA, the core genes involved in SOS responses in other bacteria, such as *recA*, *uvrA*, *ruvC*, *ruvA*, and *ruvB*, did not show significant changes in expression. The expression of other genes involved in DNA repair and recombination also did not change significantly. These findings indicate that *C. jejuni* does not mount a typical SOS response or upregulate the general DNA repair system in the early response to CIPRO treatment. Notably, *cj1085c*, a homolog of *mfd*, was upregulated in the presence of CIPRO. Two up-regulated genes, *uppP* and *uppS*, encode products involved in cell wall production [56],[57], while *pldA* encodes an outer membrane phospholipase that has been implicated in hemolysis, capsular production, and virulence [58],[59]. According to the Q values, the identified genes would have an estimated false discovery rate (FDR) of 20%. However, quantitative real-time RT-RCR (qRT-PCR*)* confirmed all of the 11 genes selected from the microarray list (Table 1), suggesting that the actual FDR is lower than the estimation.

**Table 1:** Genes differentially expressed in the presence of ciprofloxacin.

Gene ID and Functional Category		*P*-Value	*Q*-Value	*n*-Fold change	
				Microarray	qRT-PCR
**Cell membrane**					
***Cj0205***	*uppP*, undecaprenyl-diphosphatase	0.0135	0.130143	1.59	6.1
***Cj0735***	putative periplasmic protein	0.0186	0.14811	1.70	NT*
***Cj0824***	*uppS*, undecaprenyl diphosphate synthase	0.0099	0.120356	1.52	2.1
***Cj1351***	*pldA,* phospholipase A	0.0046	0.094812	2.02	2
***Cj0033***	putative integral membrane protein	0.0033	0.086471	−1.52	NT
***Cj0179***	*exbB1*, biopolymer transport protein	0.0412	0.217646	−1.88	NT
***Cj0486***	putative sugar transporter	0.0043	0.091967	−1.57	NT
***Cj0553***	putative integral membrane protein	0.0106	0.121714	−1.59	NT
***Cj0834c***	ankyrin repeat-containing possible periplasmic protein	0.0089	0.110719	−1.51	NT
***Cj1013c***	putative cytochrome C biogenesis protein	0.0058	0.096095	−1.52	NT
***Cj1662***	putative integral membrane protein	0.0055	0.096095	−1.68	NT
***Cj1663***	putative ABC transport system ATP-binding protein	0.0002	0.067622	−1.75	NT
**DNA replication, recombination and repair**					
***Cj1085c***	*mfd*, transcription-repair coupling factor	0.0029	0.082832	1.57	2.2
***Cj0718***	*dnaE*, DNA polymerase III, alpha chain	4.07E-05	0.052459	−1.62	−1.98
**Cellular process and energy metabolism**					
***Cj0041***	putative flagellar hook-length control protein	0.0357	0.204764	1.93	NT
***Cj0065c***	*folk*, putative 2-amino-4-hydroxy-6- hydroxymethyldihydropteridine pyrophosphokinase	0.0117	0.129371	1.54	NT
***Cj1030c***	*lepA*, GTP-binding protein homolog	0.0057	0.210454	1.54	NT
***Cj1280c***	putative ribosomal pseudouridine synthase	0.0252	0.170564	1.50	NT
***Cj0009***	*gltd*, glutamate synthase (NADPH) small subunit	0.0007	0.067622	−1.74	NT
***Cj0123c***	putative tRNA-dihydrouridine synthase	0.0020	0.076881	−1.87	−2.1
***Cj0227***	*argD,* acetylornithine aminotransferase	0.0208	0.151677	−1.69	NT
***Cj0283c***	*cheW*, chemotaxis protein	0.0125	0.130143	−1.52	NT
***Cj0415***	putative GMC oxidoreductase subunit	0.0191	0.148174	−1.53	NT
***Cj0490-***	*ald*, putative aldehyde dehydrogenase C-terminus	0.0013	0.076653	−1.80	NT
***Cj0537***	*oorb*, OORB subunit of 2-oxoglutarate:acceptor oxidoreductase	0.0024	0.076881	−1.67	−2.23
***Cj0734c***	*hisJ,* histidine-binding protein precursor	0.0404	0.214389	−1.59	NT
***Cj0764c***	*speA*, biosynthetic arginine decarboxylase	0.0116	0.129371	−1.97	−1.96
***Cj0767c***	*kdtB,*3-deoxy-D-manno-octulosonic-acid transferase	0.0370	0.207688	−1.58	NT
***Cj1264c***	*hydD*, putative hydrogenase maturation protease	0.0009	0.067622	−2.13	−6.7
***Cj1265c***	*hydC*, Ni/Fe-hydrogenase B-type cytochrome subunit	0.0016	0.076881	−2.12	NT
***Cj1266c***	*hydB*, Ni/Fe-hydrogenase large subunit	0.0032	0.086244	−1.56	NT
***Cj1364c***	*fumC*, fumarate hydratase	0.0255	0.171473	−1.52	NT
***Cj1476c***	pyruvate-flavodoxin oxidoreductase	0.0133	0.130143	−1.55	NT
***Cj1566c***	*nuoN*, NADH dehydrogenase I chain N	0.0039	0.088549	−2.03	−2.71
***Cj1567c***	*nuoM*, NADH dehydrogenase I chain M	0.0055	0.096095	−1.60	NT
***Cj1624c***	*sdaA*, L-serine dehydratase	0.0046	0.094812	−1.58	NT
***Cj1682c***	*gltA*, citrate synthase	0.0167	0.142007	−1.54	NT
***Cj1688c***	*secY*, preprotein translocase subunit	0.0021	0.076881	−1.63	−2.15
***Cj1717c***	*leuC*, 3-isopropylmalate dehydratase large subunit	0.0018	0.076881	−1.61	NT
**Unknown function**					
***Cj0163c***	hypothetical protein	0.0204	0.151393	1.60	NT
***Cj0814***	hypothetical protein	0.0002	0.067622	1.99	NT
***Cj0959c***	hypothetical protein	0.0233	0.160562	1.50	NT
***Cj1025c***	hypothetical protein	0.0389	0.210454	1.54	NT
***Cj0125c***	hypothetical protein Cj0125c	0.0031	0.084482	−1.53	NT
***Cj0554***	hypothetical protein	0.0083	0.107589	−1.71	NT

*: NT: Not tested.

### Characteristics of Mfd

**Figure 1:**
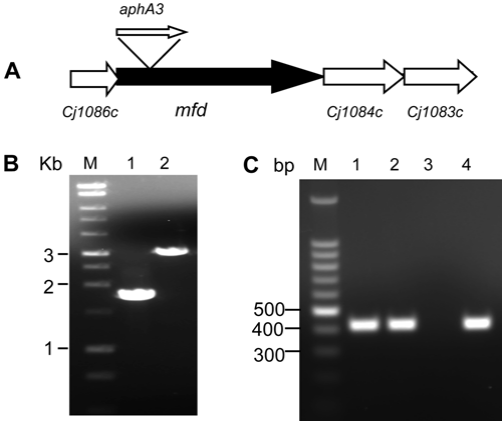
Insertional mutation of *mfd* and its impact on the transcription of *cj1084c*. (A) Diagram depicting the genomic organization of *mfd* and its flanking regions. ORFs and their directions of transcription are indicated by boxed arrows. The location of the inserted kanamycin resistance gene (*aphA3*) in *mfd* is indicated. (B) PCR confirmation of the *aphA3* insertion into the *mfd* gene in JH01. Lane 1 shows the PCR product from 11168, while Lane 2 shows the PCR product of JH01. The primers used in the PCR were mfd-F2 and mfd-R2. Lane M contains 1 kb DNA size markers (Promega). (C) RT-PCR analysis of *cj1084c* expression in strains 11168 and JH01. The same amount of total RNA from 11168 (Lane 1) and JH01 (Lane 2 and 3) were used as template in the RT-PCR. Lanes 1 and 2 are normal RT-PCR reactions. Lane 3 is a RT-PCR reaction without reverse transcriptase (DNA-free control for the RNA preparation). In Lane 4, genomic DNA of 11168 was used as template (positive control for PCR).

Cj1085c (978aa) was annotated as Mfd [48] and shows 31.5% amino acid identity to the *E. coli* Mfd protein (1148 aa). In addition, it contains the characteristic domains conserved in Mfd proteins, such as the ATP/GTP-binding site motif and the superfamily II helicase motif. Mfd in other bacteria has been shown to be involved in strand-specific DNA repair by displacing lesion-stalled RNA polymerase and recruiting enzymes involved in recombination events [54],[60]. The *mfd* locus is highly conserved in *Campylobacter* and is present in all *Campylobacter* species and *C. jejuni* strains that have been sequenced to date. The Mfd proteins in different *Campylobacter* species share 57–79% identity to the Mfd in *C. jejuni* NCTC 11168. Within *C. jejuni*, the Mfd proteins are 98–100% homologous among different strains. The *mfd* gene is located in the middle of a gene cluster, whose transcription is in the same direction (partially shown in Fig. 1A). The downstream gene *Cj1084c* encodes a putative ATP/GTP binding protein, while the upstream gene *Cj1086c* encode a hypothetical protein [48]. It is unknown if *mfd* and its flanking genes form an operon, but it appeared that *Cj1086c* and *mfd* were co-transcribed because a RT-PCR product spanning both ORFs was amplified (data not shown).

### Expression levels of *mfd* influence the frequency of emergence of spontaneous FQ-resistant mutants

**Figure 2:**
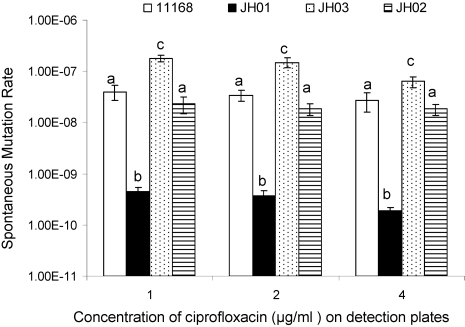
Frequencies of emergence of spontaneous FQ^R^ mutants in different *C. jejuni* strains including the wild-type 11168, the *mfd* mutant (JH01), the complemented *mfd* mutant (JH02), and the *mfd*-overexpressing construct (JH03). Three different concentrations of CIPRO (1, 2, and 4 µg/ml, respectively) were used in the detection plates to count FQ^R^ colonies. Each bar represents the mean±standard deviation of frequencies from three independent cultures. The bars labeled with different letters indicate that they are significantly different (*P*<0.05).

Since *mfd* was the only DNA repair related gene that showed a significant change in expression in the early response of *C. jejuni* to CIPRO treatment (Table 1), we examined its role in the emergence of spontaneous FQ^R^ mutants in *Campylobacter*. Firstly, the *mfd* gene was inactivated by insertional mutagenesis (Fig. 1B). As shown in Fig. 2, the *mfd* mutant (JH01) showed a approximately 100-fold reduction in the frequencies of emergence of spontaneous FQ^R^ mutants detected using plates containing three different concentrations (1, 2, and 4 µg/ml, respectively) of CIPRO. Complementation of the *mfd* mutant *in trans* by a plasmid-carried *mfd* restored the frequencies of mutant emergence to the wild-type level (JH02 in Fig. 2). As determined by qRT-PCR, the expression level of *mfd* in the complemented construct (JH02) was fully restored (1.7× the wild-type level). pRY112 alone (without the cloned *mfd* gene) did not complement the *mfd* mutant in the mutation frequency (data not shown). These results indicate that Mfd contributes significantly to the rate of spontaneous mutations to FQ resistance.

Secondly, we determined if the enhanced expression of *mfd* increases the mutation frequency. For this purpose, we constructed strain JH03, which was a wild-type 11168 strain containing an extra copy of *mfd* carried on a shuttle plasmid. In JH03, the mRNA of *mfd* increased 3.8 times compared with that in 11168 as determined by qRT-PCR. When compared with the wild-type 11168, the frequency of emergence of FQ^R^ mutants from JH03 increased about 10-fold (Fig. 2). The increase was reproducible in multiple experiments and was statistically significant (P<0.05). These results indicated that overexpression of *mfd* increases the frequency of emergence of spontaneous FQ^R^ mutants.

Given that there is only one nucleotide between the *mfd* gene and its downstream gene *cj1084c*, it was prudent to determine if the *mfd* mutation resulted in a polar effect on the expression of *cj1084c*. RT–PCR showed that *cj1084c* was transcribed at a comparable level in both the *mfd* mutant and the wild-type NCTC 11168 (Fig. 1C). RT-PCR was also performed using 10-fold serial dilutions of the RNA template, which yielded comparable results between the two strains (data not shown). PCR without the reverse transcriptase did not yield a product (Fig. 1C), indicating that the mRNA templates had no DNA contamination. These results suggested that the insertional mutation in the *mfd* gene did not cause an apparent polar effect on expression of the downstream gene. This finding plus the complementation data (Fig. 2) strongly indicate that loss of Mfd is responsible for the observed reduction in the mutation frequency in JH01.

### Loss of *mfd* does not affect the susceptibility of *C. jejuni* to antibiotics

**Figure 3:**
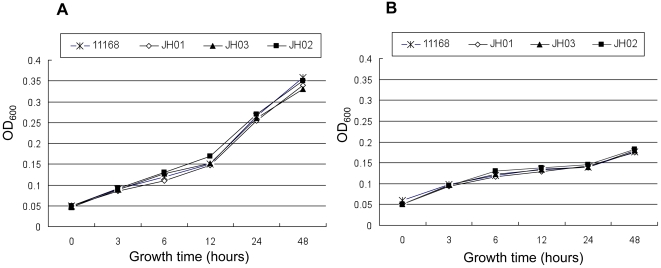
Growth kinetics of various *C. jejuni* constructs in culture media. The strains were grown in MH broth (A) or MH broth supplemented with 0.06 µg/ml of CIPRO (B).

To examine if the reduction in the emergence of spontaneous FQ^R^ mutants is caused by the increased susceptibility of the *mfd* mutant to CIPRO, we compared the MICs of several antibiotics in the *mfd* mutant with those in the wild type. Our results did not reveal any differences between the mutant and the wild type in their susceptibility to the tested antibiotics including erythromycin, ampicillin, streptomycin, and CIPRO (data not shown). In addition, there was no apparent difference in growth kinetics between the wild-type and the *mfd* mutant either in MH broth (without antibiotics) or in MH broth supplemented with a subinhibitory concentration (0.06 µg/ml) of CIPRO (Fig. 3). The growth rates of the *mfd* over-expressing strain (JH03) and the complemented mutant (JH02) were also similar to that of the wild type (Fig. 3). Thus, the reduced spontaneous mutation rate to FQ resistance in the *mfd* mutant was not attributable to decreased growth rate or increased susceptibility to antibiotics. In addition, the CIPRO-resistant colonies examined for *gyrA* mutations all carried the C257T mutation in *gyrA* and had a CIPRO MIC of >32 µg/ml regardless of the backgrounds (11168 or JH01) from which the mutants were selected.

### Mfd contributes to the emergence of FQ^R^ *Campylobacter* under *in vitro* treatment

**Figure 4:**
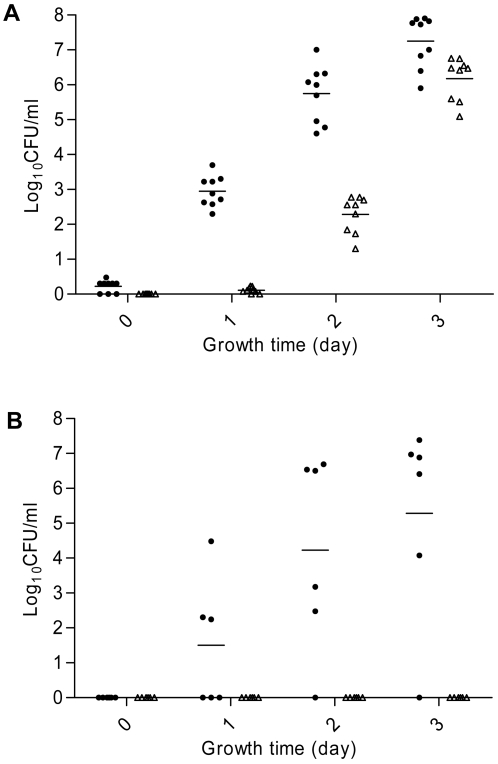
Development of FQ^R^ mutants from 11168 (solid circle) and JH01 (triangle) grown in MH broth supplemented with 4 µg/ml of CIPRO. In (A), the initial cell density (at time 0) of each culture was 10^7^ CFU/ml, while in (B) the initial cell density was 10^6^ CFU/ml. Each symbol represents the number of FQ^R^ mutants in a single culture. Each horizontal bar represents the mean log_10_ CFU/ml from each strain at a given time. (A) Displays the results of 3 independent experiments, while (B) represents the results of two independents experiments. The detection limit of the plating method is 1 CFU/ml.

FQ^R^ *Campylobacter* mutants emerge rapidly from a FQ-susceptible population once treated with FQ antimicrobials [19],[26],[27],[28],[29],[30]. To determine if Mfd influences the development of FQ^R^ *Campylobacter* under selection pressure, we conducted i*n vitro* growth experiments, in which *C. jejuni* was treated with a suprainhibitory concentrations of CIPRO (4 µg/ml). In the first treatment experiment, 10^9^ CFU of bacterial cells were inoculated into each flask containing 100 ml MH broth with 4 µg/ml of CIPRO, yielding an initial cell density of 10^7^ CFU/ml. At the beginning of the treatment, 1–3 CFU/ml of FQ^R^ mutants were detected in the flasks inoculated with 11168, while no FQ^R^ mutants were detected in the cultures inoculated with JH01 (Fig. 4A). One day after the initiation of the treatment, the numbers of FQ^R^ mutants in the 11168 cultures grew to a level ranging from a few hundreds to a few thousands CFU/ml, while no mutants or about 1 CFU/ml of FQ^R^ mutants were detected in the cultures of JH01 (Fig. 4A). The FQ^R^ populations expanded on day 2 in both strains, but the FQ^R^ population of JH01 was still about 1,000-fold less than that of 11168. Due to the continued enrichment of the FQ^R^ mutants by CIPRO and the fact that the mutants of 11168 was entering the stationary phase, the average difference between 11168 and JH01 on day 3 decreased, but was still more than one order of magnitude (Fig. 4A). In the second experiment, 2×10^7^ CFU bacterial cells of 11168 or JH01 were inoculated into each flask containing 20 ml of MH broth with 4 µg/ml of CIPRO, yielding an initial cell density of 10^6^ CFU/ml. At the beginning of the treatment, no FQ^R^ mutants were detected from either 11168 or JH01 (Fig. 4B). On day 1 after the initiation of the treatment, FQ^R^ *Campylobacter* emerged from some of the cultures of 11168 and continued to expand in numbers on day 2 and day 3. In contrary to 11168, no FQ^R^ mutants emerged from any of the JH01 cultures during the three-day incubation (Fig. 4B). In the third experiment, the inoculum was decreased to 2×10^4^ CFU per flask (initial cell density = 10^3^ CFU/ml), and no FQ^R^ mutants were detected from either 11168 or JH01 after three day's incubation (data not shown). These results indicated that emergence of FQ^R^ mutants under treatment with CIPRO was influenced by the initial bacterial cell density and facilitated by the function of Mfd.

### Mfd affects the emergence of FQ^R^ mutants *in vivo*

**Figure 5:**
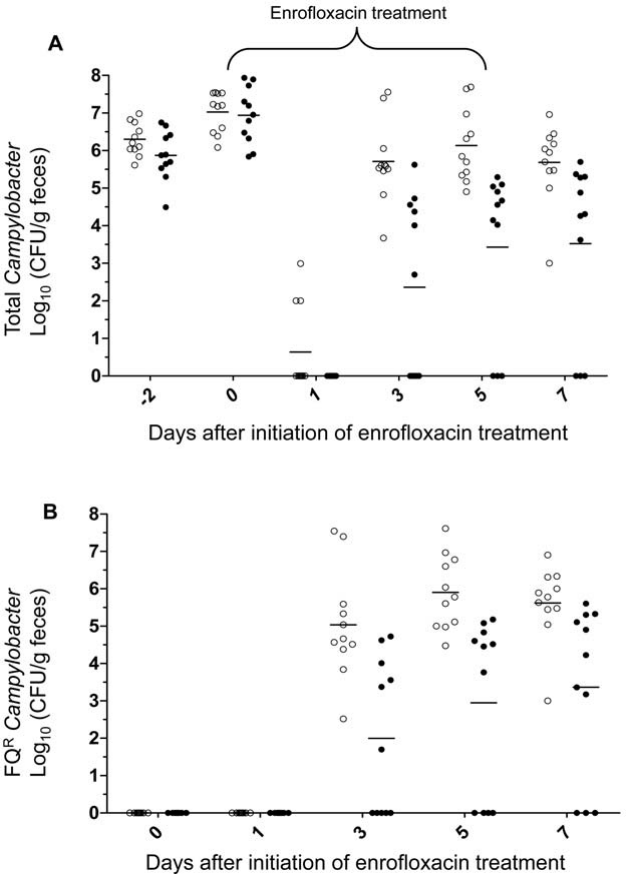
Development of FQ^R^ *Campylobacter* mutants in chickens initially infected with FQ-susceptible *Campylobacter*, but treated with enrofloxacin. (A) The level of total *Campylobacter* in each chicken inoculated with the wild-type 11168 (open circle) or the *mfd* mutant strain (JH01; solid circle). The treatment with enrofloxacin started on day 0 and lasted for five consecutive days (indicated by a bracket on top of the panel). (B) The level of FQ^R^ *Campylobacter* in each chicken inoculated with the wild-type (open circle) or the *mfd* mutant (solid circle). In both panels, each symbol represents the number of *Campylobacter* in a single bird. Each group includes eleven chickens and the mean of each group at a given time is indicated by a horizontal bar. A chicken is considered negative if the level of colonization was below the detection limit (10^2^ CFU/ g of feces).

To determine if Mfd influences the emergence of FQ^R^ *Campylobacter* during *in vivo* therapeutic treatment, broiler chickens were infected with 11168 or JH01 and then treated with enrofloxacin administered in drinking water (50 ppm). The birds in both groups were quickly colonized by *C. jejuni* after inoculation (Fig. 5). Before the treatment with enrofloxacin, all birds were colonized by *Campylobacter* and the colonization levels (CFU/g feces) were similar in both groups (*p*>0.05). One day after initiation of the treatment, the number of colonized chickens and the levels of colonization decreased drastically in both groups, with *Campylobacter* detectable only in three chickens that were inoculated with the wild-type strain (Fig. 5A). After that, the numbers of *Campylobacter* in both groups rebounded. On day 3 after the initiation of the treatment, all of the birds in the 11168 group were re-colonized by *Campylobacter* and remained colonized until the end of the experiment. For the group inoculated with JH01, 6 of the11 birds became positive with *Campylobacter* on day 3 after initiation of the treatment (Fig. 5A) and 3 birds remained negative until the end of the experiment. On days 3, 5 and 7 after initiation of the treatment, the average colonization level of the JH01-inoculated group was approximately 3 log units lower than that of the 11168-inoculated group (Fig. 5A) and the differences were statistically significant (*p*<0.05). The number of FQ^R^ *Campylobacter* in each chicken was also monitored. Prior to the treatment, no FQ^R^ *C. jejuni* was detected in any of the chickens (Fig. 5B). On day 1 after initiation of the treatment, the three *Campylobacter-*positive birds of the 11168-inoculated group still carried FQ-susceptible *Campylobacter*. However, FQ^R^ *C. jejuni* appeared on day 3 in all birds of the 11168-inoculated group and in some birds of the JH01-inoculated group (Fig. 5B). Comparison of the total *Campylobacter* counts (Fig. 5A) with the numbers of FQ^R^ *Campylobacter* (Fig. 5B) revealed that the birds were re-colonized by FQ^R^ mutants after initiation of the treatment. The average numbers of FQ^R^ *Campylobacter* in the JH01-inoculated group were approximately 3 log units lower that those of the 11168-inoculated group (Fig. 5B) and the differences were statistically significant (*p*<0.05). These results indicate that loss of Mfd significantly reduced the rates of emergence of FQ^R^ *Campylobacter* in enrofloxacin-treated chickens.

Representative *Campylobacter* isolates obtained at different sampling times from both groups were tested for CIPRO MICs using E-test strips. The result showed that before treatment all the tested isolates from both groups were susceptible to CIPRO (MICs = 0.094–0.125 µg/ml). The majority of the tested isolates from day 1 after initiation of the treatment were still susceptible to CIPRO (MICs = 0.094–0.5 µg/ml). On day 3 after the initiation of treatment, 21 of the 22 tested isolates (from both groups) had a CIPRO MIC of >32 µg/ml and the other one had an MIC of 8 µg/ml. Similarly, the majority (44 out of 49) of the tested isolates from days 5 and 7 had a CIPRO MIC of >32 µg/ml and the rest had MICs from 1–24 µg/ml. The MIC results further confirmed the differential plating results that the chickens were re-colonized by FQ^R^ *Campylobacter*.

## Discussion

When *Campylobacter* cells were treated with a subinhibitory concentration (0.06 µg/ml, 0.5× the MIC) of CIPRO for 1.5 hours, no significant changes in gene expression were detected using the cut-off criteria defined in this study. This result was somewhat similar to the study with *Haemophilius influenzae* [61] in that the treatment with a low concentration of CIPRO induced few changes in gene expression, but was different from that study because several genes involved in SOS response were upregulated in *Haemophilius influenzae*. Prolonged treatment of *Campylobacter* with the subinhibitory concentration of CIPRO may reveal noticeable changes in gene expression, but culturing *Campylobacter* with 0.06 µg/ml of CIPRO reduces its growth rate (Fig. 3), which will make the comparison with the non-treated control unfeasible and complicate the interpretation of the microarray results. To mimic clinical treatment, *C. jejuni* cells were exposed to a suprainhibitory dose (1.25 µg/ml, 10× the MIC) of CIPRO. This dose is within the concentration range of CIPRO in gut contents during FQ treatment in chickens [62]. The reason that we treated the samples for 0.5 hour instead of a longer time was to detect the primary response triggered by CIPRO, instead of the secondary response caused by cell death. When *Campylobacter* cells were treated with this suprainhibitory dose for 0.5 hour, the expression of multiple genes was significantly altered (Table 1). Notably, the majority of the affected genes were downregulated and many of them are involved in cellular processes and energy metabolism (Table 1). This result is similar to the findings obtained with other bacteria [43],[44],[61] and supports the notion that reducing cellular metabolism is a common strategy utilized by bacteria to cope with antibiotic treatment.

Within bacterial cells CIPRO interacts with gyrase and DNA, blocking DNA replication and transcription [18]. When exposed to CIPRO, the expression of *gyrA* and *gyrB* in various bacteria was either altered or unchanged [43],[61],[63]. In this study, we found that the expression of *gyrA*, *gyrB*, and *topA* was not significantly affected in *Campylobacter* by CIPRO. In addition, the expression of the genes encoding enzymes involved in DNA repair, recombination, or mutagenesis, such as *recA, ruvABC, uvrABC*, and *mutS*, did not change significantly. Only two genes involved in DNA metabolism (*mfd* and *dnaE*) were affected by CIPRO under the conditions used in this study (Table 1). Theses observations indicate that *C. jejuni* does not mount a typical SOS response under the treatment with FQ. These findings are also consistent with the fact that *C. jejuni* lacks LexA, the key regulator of bacterial SOS responses [46].

In addition to transcription-coupled DNA repair, Mfd has been associated with other functions in bacteria [64]. For example, Mfd of *Bacillus subtilis* is involved in homologous DNA recombination and stationary-phase mutagenesis [65],[66]. Inactivation of the *mfd* gene of *B. subtilis* resulted in a great reduction in the number of prototrophic revertants to Met^+^, His^+^, and Leu^+^ during starvation [66], indicating that Mfd promotes adaptive mutagenesis. This finding is in contrast to the known function of Mfd in mediating mutation frequency decline and could be explained by the role of Mfd in promoting transcriptional bypass and consequently increasing the adaptive mutagenesis rates [66].

In this study we found that Mfd increases the frequency of emergence of spontaneous FQ^R^ mutants in *Campylobacter* (Fig. 2). Furthermore, the *mfd* mutation also decreased the frequency of emergence of spontaneous streptomycin-resistant mutants in *Campylobacter* (data not shown). Together, the results convincingly showed that Mfd is an important player in modulating the mutation rates in *Campylobacter*. To our knowledge, this is the first report documenting the key role of Mfd in promoting spontaneous mutation rates in a bacterial organism. How Mfd contributes to the increased mutation rates in *Campylobacter* is unknown, but it can be speculated that transcriptional bypass mediated by Mfd may actively occur in replicating non-stressed *Campylobacter* populations, resulting in an elevated level of retromutagenesis (fixed changes in DNA sequence due to transcriptional mutation [67]) that contributes to the size of the mutant pools. This possibility remains to be examined in future studies. Although *mfd* contributes significantly to the mutation rate (Fig. 2), its expression level was not precisely proportional to the mutation frequencies. For example, expression of *mfd* was upregulated 3.8-fold in JH03, but its mutation frequency increased 10-fold. This difference is probably due to the fact that emergence of spontaneous mutants is a multi-step process and Mfd only contributes to one of the steps in the process. It is also possible that Mfd interacts with other proteins in modulating the mutation frequency. Thus, the changes in *mfd* expression level and the mutation frequency are not exactly at the same scale.

Another interesting observation of this study is the upregulation of *mfd* by CIPRO. The enhanced expression may be needed for transcription repair because CIPRO treatment causes DNA damage, which stalls RNA polymerase. Alternatively, the increased production of Mfd may enhance transcriptional bypass of the non-repaired DNA lesions in order to maintain cell viability and/or promote mutations for resistance. This possibility is high given the facts that massive DNA damage incurred by a suprainhibitory dose of CIPRO may overwhelm the DNA repair system and *Campylobacter* must maintain certain levels of transcription to survive the treatment, that Mfd contributes significantly to the mutation rates to FQ resistance (Fig. 2), and that *Campylobacter* does not have the error-prone DNA polymerases, such as Pol II, Pol IV, and Pol V [48]. *E. coli* and other bacteria have these error-prone DNA polymerases [68],[69], which are repressed by LexA, but upregulated by the SOS response triggered by DNA damage. Once produced, the enzymes perform translesion DNA synthesis, allowing replication to continue without DNA repair. This special functional feature results in reduced genetic fidelity, but allows for bacterial survival under stress. The outcome of the enhanced expression of the error-prone enzymes is the increased mutation rates, which contribute to the emergence of drug resistance [70]. In the absence of a SOS response and the error-prone DNA polymerases, *Campylobacter* may use Mfd as an alternative pathway to increase mutation rates. Thus, enhanced expression of *mfd* may represent an adaptive response of *Campylobacter* to the stresses imposed by CIPRO treatment. How CIPRO upregulates *Campylobacter* Mfd is unknown and further work in this direction is warranted.

FQ^R^ *Campylobacter* readily emerges from a FQ-susceptible population when treated with FQ antimicrobials (Figs. 4 and 5). As shown in the *in vitro* experiment, the development of FQ^R^ population under CIPRO treatment is influenced by the initial cell density (Fig. 4 and the corresponding text) as well as the functional state of Mfd. Considering the differences in spontaneous mutation rate between 11168 and JH01 (Fig. 2), it was likely that the 11168 and JH01 inocula had different numbers of pre-existing FQ^R^ mutants, which were selected by CIPRO and contributed to the differences in the FQ^R^ population detected in the cultures of the two strains. The inoculum-dependent emergence of FQ^R^ mutants in both 11168 and JH01 suggests that development of FQ^R^ *Campylobacter* under FQ treatment involves selection of preexisting mutants. However, the magnitude and dynamics of FQ^R^ development can not be totally explained by selection. For example, in some cultures FQ^R^ mutants were not detectible until the 2^nd^ day of the incubation (Fig. 4). A single mutant at time zero in a culture flask would grow to a population of more than 2,000 cells in one day (the generation time of *C. jejuni* in MH broth is about 2 hours), which would be readily detected by the plating method on day 1. Thus, if selection was the only factor in the development of FQ^R^ *Campylobacter*, the latest time for detecting the pre-existing mutants in the mutant-positive flasks would be day 1 after initiation of the treatment. Obviously, this was not the case for all of the cultures because some of them did not show FQ^R^ mutants until day 2 (Fig. 4). In addition, some cultures were negative with FQ^R^ mutants at time zero, but showed a large number of mutants at day 1, which could not be easily explained by sole selection of a few preexisting mutants from the inocula. Considering these unexplainable observations and the fact that a small fraction of the FQ-susceptible inoculum survived the killing effect as long as one day after the initiation of the treatment (data not shown), it was possible that new FQ^R^ mutants were developed during the treatment. If this occurs, Mfd may enhance the emergence of new mutants by promoting transcriptional bypass or other mechanisms, which may partly explain the differences between 11168 and JH01 in the dynamics of emergence of FQ^R^ mutants. Thus, there is a possibility that both selection of pre-existing mutants and de nova formation of mutants are involved in the development of FQ^R^ *Campylobacter* during treatment with FQ antimicrobials.

The role of Mfd in the development of FQ^R^ mutants was further shown by the *in vivo* experiment, in which *Campylobacter*-infected chickens were treated with enrofloxacin (Fig. 5). Previous studies have shown that therapeutic use of FQ antimicrobials in chickens promotes the emergence of FQ^R^ *Campylobacter* [19],[27],[28],[29],[30], which can be potentially transmitted to humans via the food chain. In this study, we showed that inactivation of *mfd* significantly reduced the development of FQ^R^ *Campylobacter* in chickens (Fig. 5). In fact, several birds in the JH01-inoculated group became negative with *Campylobacter* once the treatment was initiated. Since the *mfd* mutant did not show a growth defect *in vitro* (Fig. 3) and colonized chickens as efficiently as the wild-type strain (see the colonization level before treatment in Fig. 5), the observed differences in the development of FQ^R^ mutants were not due to changes in growth characteristics. These *in vivo* results (Fig. 5) plus the *in vitro* findings (Fig. 4) clearly showed that Mfd plays an important role in the development of FQ^R^ *Campylobacter* mutants under the selection pressure. To our knowledge, this is the first report that documents the role of Mfd in the development of FQ resistance in a bacterial pathogen. Since Mfd is highly conserved in bacterial organisms [64], it would be interesting to know if this finding applies to other bacterial pathogens. In addition, inhibition of Mfd functions may represent a feasible approach to reducing the emergence of FQ^R^ *Campylobacter*.

## Materials and Methods

### Bacterial strains and growth conditions

*C. jejuni* strain NCTC 11168 (ATCC 700819) was used in this study. The strain was routinely grown in Mueller-Hinton (MH) broth (Difco) or on MH agar at 42°C under microaerobic conditions (5% O_2_, 10% CO_2_, and 85% N_2_). The media were supplemented with kanamycin (50 µg/ml) or chloramphenicol (4 µg/ml) as needed. *Escherichia coli* cells were grown at 37°C with shaking at 200 r.p.m. in Luria Bertani (LB) medium which was supplemented with ampicillin (100 µg/ml) or kanamycin (30 µg/ml) when needed.

### DNA microarray and qRT-PCR

DNA microarray was used to identify genes that were differentially expressed in *C. jejuni* 11168 treated with CIPRO. For RNA isolation, *Campylobacter* cells were grown for 24 hours to the mid exponential phase (OD_600_≈0.1∼0.15) and split into two equal portions, one of which was treated with CIPRO and the other served as a non-treated control. A subinhibitory concentration (0.06 µg/ml, 0.5× the MIC) and a suprainhibitory dose (1.25 µg/ml, 10× the MIC) of CIPRO were used in the treatments. For the treatment with 0.06 µg/ml of CIPRO, the treated and non-treated samples were incubated at 42°C for 1.5 hours under microaerobic conditions, while for the treatment with 1.25 µg/ml of CIPRO, the samples were incubated at 42°C for 30 min under microaerobic conditions. Immediately after the incubation, RNAprotect Bacteria Reagent (Qiagen, Valencia, CA) was added to the cultures to stabilize mRNA. The total RNA from each sample was extracted using the RNeasy Mini Kit (Qiagen). The purified RNA samples were treated with On-Column DNase Digestion Kit (Qiagen) followed by further treatments with DNase to remove residual DNA contamination. RNA samples were extracted from 6 independent treatments with each concentration of CIPRO. Absence of contaminating DNA in the RNA samples was confirmed by RT-PCR. The concentration of total RNA was estimated with the NanoDrop ND-1000 spectrophotometer (NanoDrop, Wilmington, DE, USA), and the integrity and size distribution of the purified RNA was determined by denaturing agarose gel electrophoresis and ethidium bromide staining. The quality of total RNA was further analyzed using the Agilent 2100 Bioanalyzer (Agilent Technologies, Santa Clara, CA, USA), which showed the good quality and integrity of the RNA samples (Data not shown).

cDNA synthesis and labeling, microarray slide (Ocimum Biosolutions) hybridization, Data collection and normalization, and statistical analysis were performed as described in a previous publication [71]. For each type of treatment (0.06 µg/ml for 1.5 hours or 1.25 µg/ml for 30 min), six microarray slides were hybridized with RNA samples prepared from 6 independent experiments. For this study, we chose *p-*value<0.05 and the change ≥1.5-fold as the cutoff for significant differential expression between the treated and non-treated samples. Representative genes identified by the DNA microarray were further confirmed by qRT-PCR as described in a previous work [72]. The primers used for qRT-PCR are listed in Table 2.

**Table 2:** Oligonucleotide primers used in qRT-PCR.

Primer	Sequence	Gene amplified
16s RNA F	5′-TAC CTG GGC TTG ATA TCC TA-3′	16s RNA
16s RNA R	5′-GGA CTT AAC CCA ACA TCT CA-3′	*cj0123c*
Cj0123cF	5′ CGC CTT GAT CTT TGT AGT GTT TT 3′	
Cj0123cR	5′ TGA AAT CAA AAG CGG TAA AAG TG 3′	
Cj0824F1	5′-CAA AGT GCG TCA CAA TGC TT-3′	*cj0824 (uppS)*
Cj0824R1	5′-GAT TTA TCG CGC TTG GAA GA-3′	
Cj1351F1	5′-ATC CCC TTG GCA TTA GCT CT-3′	*cj1351 (pldA)*
Cj1351R1	5′-TGG AAT TTC GCC TCA CTA TT-3′	
Cj1264cF1	5′-GCT TAG GCG TTC ATC TTT GC-3′	*cj1264c*
Cj1264cR1	5′-CAA AGC CAA AGT TCC ACC AT-3′	
Cj1085cF1	5′-TGT TTT GCA AAC TCC ACC AG-3	*cj1085c (mfd)*
Cj1085cR1	5′-ATT TTG CCC ACC ACG TCT TA-3′	
Cj0205F1	5′-GAA AAG TTG CGG CTG AGT TT-3′	*cj0205 (uppP)*
Cj0205R1	5′-AAT TTG CAT TGC CAA GAA GC-3′	
Cj0537F1	5′-GGC AAT TGG TGG AAA TCA TAC TA-3′	*cj0537*
Cj0537R1	5′-TGG AGT AGT TGG AGA AGT TTG AGA-3′	*cj0718(dnaE)*
Cj0718F	5′ GGACTTGGGGCTATAAAAAGTGT 3′	
Cj0718R	5′ GGACTTGGGGCTATAAAAAGTGT 3′	
Cj1688cF1	5′-GCC TGA ATT GAT TTG TCC TAC AG-3′	*cj1688c* (*secY*)
Cj1688cR1	5′-CGA ACA AAT CAC ACA AAG AGG TA-3′	
Cj0764cF1	5′-TTC AGC TGC AAT AAA GCC TAT GT-3′	*cj0764c (speA*)
Cj0764cR1	5′-ATA ATA ACG AAG GCG CAC CTA TT-3′	
Cj1566cF1	5′-CAT AAA TTT ACC CCA AAA CAC TCC-3′	*cj1566c*
Cj1566cR1	5′-GAG AGT TTA AAT GGG CTT TTG GT-3′	

### Insertional mutation of *mfd*

An isogenic *mfd* (*cj1085c*) mutant of strain NCTC 11168 was constructed by insertional mutagenesis. Primers mfd-F2 (5′-TGTTGATGGAGAGTTAAGTGGTAT-3′) and mfd-R2 (5′-AATAGCATTCATAGCGACTTCTGTT-3′) were designed from the published genomic sequence of this strain [48] and used to amplify a 1.8-kb fragment spanning the 5′ region of *mfd*. Amplification was performed with *Pfu Turbo* DNA Polymerase (Stratagene, La Jolla, CA, USA). The blunt-ended PCR product was purified using a QIAquick PCR purification kit (Qiagen, Valencia, CA, USA), ligated to *Sma*I–digested suicide vector pUC19, resulting in the construction of pUC-mfd, which was then transformed into *E. coli* DH5α. Since a unique *EcoR*V site (which generates blunt ends) occurs in the cloned *mfd* fragment, pUC-mfd was digested with *EcoR*V to interrupt the *mfd* gene. Primers KanNco-F (5′ CTT ATC AAT ATA TCC ATG GAA TGG GCA AAG CAT 3′) and KanNco-R (5′ GAT AGA ACC ATG GAT AAT GCT AAG ACA ATC ACT AAA 3′) were used to amplify the *aphA3* gene (encoding kanamycin resistance) from the pMW10 vector [73] by using *Pfu Turbo* DNA polymerase (Stratagene). The *aphA3* PCR product was directly ligated to *EcoRV*-digested pUC-mfd to obtain construct pUC-mfd-aphA3, in which the *aphA3* gene was inserted within *mfd* (the same direction as the transcription of *mfd*) and the insertion was confirmed by PCR using primers mfd-F2 and Kana-intra (5′ GAA GAA CAG TAT GTC GAG CTA TTT TTT GAC TTA 3′). The pUC-mfd-aphA3 construct, which served as a suicide vector, was electroporated into *C. jejuni* NCTC 11168. Transformants were selected on MH agar containing 10 µg/ml of kanamycin. Inactivation of the *mfd* gene in the transformants by insertion of the *ahpA3* gene was confirmed by PCR using primers mfd-F2 and mfd-R2 (Fig. 1B). The *mfd* mutant of NCTC 11168 was named JH01.

### Complementation of the *mfd* mutant *in trans*

The entire *mfd* gene including its putative ribosome binding site was amplified from strain 11168 by PCR using primers mfd-F5 (5′-CGCTTCCGCGGAACTAGTAAAATTAAAGAAGATACTATC-3′) and mfd-R3 (5′-GGCTTTAAATAATCTTTTCGAGCTCTATAAATT-3′). The underlined sequences in the primers indicate the restriction sites for *Sac*I and *Sac*II, respectively. The PCR product was digested with *Sac*I and *Sac*II, and was then cloned into the plasmid construct pRY112-pABC to generate pRY112-mfd, in which the *mfd* gene was fused to the promoter of *cmeABC*. pRY112-pABC was made by cloning the promoter sequence of *cmeABC* [74] to shuttle plasmid pRY112 [75]. The promoter DNA of *cmeABC* was amplified by primers BSF (5′ AAAAGGATCCTAAATGGAATCAATAG 3′) and AR2 (5′ TGATCTAGATCATACCGAGA 3′), digested with *Bam*HI and *Xba*I, and cloned into pRY112. There were two reasons that we used the promoter of *cmeABC* in the expression of *mfd*. First, the 5′ end of *mfd* overlaps with its upstream gene and the native promoter for *mfd* was unknown. Second, the promoter of *cmeABC* is moderately active in *Campylobacter* [74], preventing over- or under-expression of *mfd*. The constructed plasmid pRY112-mfd was sequenced and confirmed that no mutations in the cloned sequence occurred. For complementation, the shuttle plasmid pRY112-mfd was transferred into JH01 by conjugation. The complemented strain was named JH02. Limited passage of JH02 in MH broth without antibiotics indicated that the complementing plasmid was stable in the construct (data not shown). The shuttle plasmid carrying the *mfd* gene was also transferred to wild-type 11168 to generate strain JH03 for overexpression of the *mfd* gene.

### Growth rates in MH broth with or without CIPRO

To compare the growth kinetics of the *mfd* mutant with that of the wild-type, a fresh culture of each strain was inoculated into MH broth (initial cell density of OD600 = 0.05) and the cultures were incubated at 42°C under microaerobic conditions. To determine if the mutation affects *C. jejuni* growth with a subinhibitory concentration of CIPRO, the various strains were grown in MH broth with 0.06 µg/ml of CIPRO (0.5× the MIC). Culture samples were collected and measured for OD_600_ at 0, 3, 6, 12, 24 and 48 hours post-inoculation.

### Antibiotic susceptibility test

The minimum inhibitory concentration (MIC) of CIPRO was determined by using E-test strips (AB Biodisk, Solna, Sweden) as described in the manufacturer's instructions. The detection limit of the E-test for CIPRO was 32 µg/ml. The MICs of erythromycin, ampicillin and streptomycin for *C. jejuni* NCTC 11168, JH01, JH02, and JH03 were determined using a standard microtiter broth dilution method described previously [24]. Each MIC test was repeated at least three times to confirm the reproducibility of the MIC patterns. The antibiotics used in this study were purchased from Sigma Chemical Co. (erythromycin, ampicillin, streptomycin) or ICN Biomedicals Inc. (CIPRO).

### Frequencies of emergence of spontaneous FQ^R^ mutants *in vitro*

Wild-type 11168, JH01, JH02 and JH03 were compared for the spontaneous mutation rates to CIPRO resistance. In each experiment, each of the 4 strains was inoculated into three flasks, each of which contained 30 ml of antibiotic-free MH broth. The cultures were incubated to the mid logarithmic phase (OD_600_≈0.15) under microaerobic conditions. The culture in each flask was collected by centrifugation and resuspended in 1 ml of MH broth. The total CFU in each culture was measured by serial dilutions and plating on MH agar plates, while the number of FQ^R^ mutants was detected using MH agar plates containing 1, 2 or 4 µg/ml CIPRO. The frequency of emergence of FQ^R^ mutants was calculated as the ratio of the CFU on CIPRO-containing MH agar plates to the CFU on antibiotic-free MH agar plates after 2 days of incubation at 42°C under microaerobic conditions. This experiment was repeated five times. The mutation frequency data were log-transformed for statistical analysis. One-Way ANOVA followed by Tukey test was used to determine the significance of differences in the levels of spontaneous mutation rates among the strains. The data were also analyzed by the Wilcoxon rank-sum test to allow for non-normality. For the comparisons discussed in Results, the conclusion of the two tests was the same at significance level 0.05.

### Sequence analysis of the QRDR of *gyrA*

Representative FQ^R^ colonies were selected for determination of the point mutations in *gyrA*. The QRDR of *gyrA* was amplified by PCR using primer pair GyrAF1 (5′-CAACTGGTTCTAGCCTTTTG-3′) and GyrAR1 (5′-AATTTCACTCATAGCCTCACG-3′) [76]. The amplified PCR products were purified with the QIAquick PCR purification kit (Qiagen) prior to sequence determination. DNA sequence analysis was carried out using an automated ABI Prism 377 sequencer (Applied Biosystems, Foster City, CA, USA) and analyzed by the Omiga 2.0 (Oxford Molecular Group) sequencing analysis software.

### *In vitro* treatment with CIPRO

To determine if Mfd affects the development of FQ^R^ mutants under treatment with CIPRO, wild-type 11168 and JH01 were treated in MH broth with 4 µg/ml (32× the MIC) of CIPRO. Wild-type 11168 and JH01 were grown on antibiotic-free MH agar plates under microaerobic conditions. After 20 hours of incubation, the cells were collected and resuspended in MH broth for inoculation. Three treatment experiments were conducted using three different initial cell densities. In experiment 1, each strain was inoculated into 3 100-ml flasks with MH broth containing 4 µg/ml of CIPRO and the initial cell density was 10^7^ CFU/ml. The cultures were incubated microaerobically at 42°C. Aliquots of the cultures were collected at different time points (0, 1, 2, 3 days post-inoculation) and plated onto regular MH plates for enumeration of the total bacterial number and onto MH plates containing 4 µg/ml of CIPRO for counting FQ^R^ colonies. In experiments 2 and 3, the cultures were treated in the same way, but the initial cell densities were 10^6^ and 10^3^ CFU/ml, respectively. Experiment 1 was repeated three times, while experiments 2 and 3 were each repeated twice.

### The transcription level of *Cj1084c*

To determine if the insertional mutation in *mfd* affected the expression of the downstream gene *Cj1084c* (encoding a possible ATP/GTP-binding protein), RT-PCR was performed to assess the expression of *Cj1084c.* Total RNA was isolated from *C. jejuni* 11168 and JH01 using the RNeasy Kit (Qiagen). The purified RNA samples were treated with On-Column DNase Digestion Kit (Qiagen) followed by further treatments with DNase to remove DNA contamination. The *Cj1084c*-specific primers Cj1084cF (5′ TTG CCT TAG CAG ATA TCA T 3′) and Cj1084cR (5′ ACC ACT TCT ACT TGC TCT TA 3′) were used to amplify a 430 bp region of the gene in a conventional one-step RT-PCR by using the SuperScript III One-Step RT-PCR kit (Invitrogen). An RT-PCR mixture lacking the RT was included as a negative control.

### Emergence of FQ^R^ mutants in enrofloxacin-treated chickens

To examine if Mfd plays a role in the emergence of FQ^R^ *Campylobacter* during *in vivo* FQ treatment, a chicken experiment was performed using 11168 and JH01. Day-old broiler chickens (Ross×Cobb) were obtained from a commercial hatchery and randomly assigned to 2 groups (11 birds per group). Each group of chickens was maintained in a sanitized wire-floored cage. Feed and water were provided *ad libitum*. Prior to inoculation with *Campylobacter,* the birds were tested negative for *Campylobacter* by culturing cloacal swabs. At day 3 of age, the two groups of chickens were inoculated with 11168 and JH01, respectively, at a dose of 10^6^ CFU/chick via oral gavage. Six days after the inoculation, the birds were treated with 50 ppm enrofloxacin. The treatment was administered in drinking water and lasted for five consecutive days. During the treatment, only medicated water was given to the birds to ensure enough consumption. Cloacal swabs were collected periodically before and after enrofloxacin treatment until the end of the experiment. Each swab was serially diluted in MH broth and plated onto two different types of MH plates: one containing *Campylobacter*-specific growth supplements (SR 084E and SR117 E; Oxoid Ltd., Basingstoke, England) for the enumeration of total *Campylobacter* cells and the other containing 4 µg/ml of CIPRO in addition to the same selective agents and supplements to recover FQ^R^ *Campylobacter* in each chicken. At each sampling time, at least one *Campylobacter* colony from each chicken were selected from the regular MH agar plates (no CIPRO) for the determination of CIPRO MICs using the E-test (AB Biodisk). The colonization data (CFU/g feces) were log-transformed and used for statistical analysis. The significance of differences in the level of colonization between the two groups was determined using Student's t-test, Welch's *t*-test to allow for non-constant variation across treatment groups, and the Wilcoxon rank-sum test to allow for non-normality. The conclusion of all three tests was the same at significance level 0.05.

### Microarray data accession number

The microarray data have been deposited in the NCBI Gene Expression Omnibus (GEO; http://www.ncbi.nlm.nih.gov/geo/) database and the accession number is GSE10471.
